# Smart Theranostic Nanoplatforms in Oral Squamous Cell Carcinoma: Integrating Diagnosis, Targeted Therapy and Synergistic Photothermal/Photodynamic Approaches

**DOI:** 10.3390/ijms27188292

**Published:** 2026-09-17

**Authors:** Ludovica Lucchesi Palli, Cinzia Scialabba, Gennara Cavallaro

**Affiliations:** Laboratory of Biocompatible Polymers, Department of Biological, Chemical and Pharmaceutical Sciences and Technologies (STEBICEF), University of Palermo, Via Archirafi 32, 90123 Palermo, Italy; ludovica.lucchesipalli@unipa.it (L.L.P.); cinzia.scialabba@unipa.it (C.S.)

**Keywords:** oral squamous cell carcinoma (OSCC), nanotheranostics, multifunctional nanoparticles, photodynamic therapy (PDT), photothermal therapy (PTT), molecular imaging, targeted drug delivery

## Abstract

Oral squamous cell carcinoma (OSCC) is the most common malignant tumor of the oral cavity and is still associated with a poor prognosis today. Although surgery, radiotherapy, chemotherapy, and more recently immunotherapy and targeted therapies have improved the clinical management of the disease, their efficacy is often limited by poor selectivity, systemic toxicity, and the development of therapeutic resistance. In this context, nanotheranostics has emerged as one of the most promising strategies in the field of precision medicine, thanks to the development of multifunctional nanoplatforms capable of integrating diagnostic and therapeutic functions within a single system. In addition to improving the delivery and controlled release of anticancer agents, these nanosystems can enable real-time monitoring of biodistribution and therapeutic response through integrated imaging techniques, supporting imaging-guided and personalized treatment. In particular, the integration of photothermal therapy (PTT) and photodynamic therapy (PDT) with other therapeutic strategies has led to the development of increasingly multifunctional platforms capable of exploiting synergistic mechanisms to enhance antitumor efficacy. This review provides a state of overview of the major theranostic nanoplatforms developed for the treatment of OSCC, critically analyzing lipid, polymeric, inorganic, carbon-based, biomimetic, and multifunctional hydrogel-based nanosystems, with a focus on targeting strategies, various integrated imaging modalities, stimulus-responsive drug delivery systems, and PTT/PDT-based combination therapies.

## 1. Introduction

Oral squamous cell carcinoma (OSCC) is the predominant histological type of oral malignancy and continues to represent a major clinical challenge worldwide [1]. According to GLOBOCAN 2022 estimates, approximately 390,000 new cases and 190,000 deaths from lip and oral cavity cancer occurred worldwide in 2022 [2]. Although considerable progress has been made in both diagnosis and treatment over the past decades, patient outcomes remain unsatisfactory [1]. This is largely ascribable to the aggressive nature of the disease, its strong tendency to invade surrounding tissues and metastasize, and the fact that many patients are still diagnosed at advanced stages. Furthermore, conventional treatment options are often limited by poor tumor selectivity, systemic toxicity, and the emergence of drug resistance. While targeted therapies and immunotherapy have broadened the therapeutic landscape, their overall clinical benefit is still modest, emphasizing the need for more effective and personalized treatment strategies [3].

In this context, nanomedicine has emerged as a promising approach for overcoming some of the limitations associated with conventional cancer therapies. Rather than acting simply as drug carriers, advanced nanosystems can combine multiple therapeutic and diagnostic functions within a single platform, giving rise to the concept of nanotheranostics [4]. Their small size, together with the possibility of tailoring their composition, morphology, and surface chemistry, can improve drug stability, prolonged circulation, selective accumulation at the tumor site, and controlled payload release in response to tumor-associated stimuli or external triggers. At the same time, the incorporation of imaging capabilities allows non-invasive monitoring of nanoparticle biodistribution and therapeutic response, paving the way for image-guided therapeutic strategies that are increasingly aligned with the principles of precision medicine [5].

In the specific case of OSCC, these strategies are particularly promising, partly due to the anatomical accessibility of the tumor. In fact, the easy accessibility of the oral cavity allows not only for direct monitoring of lesions but also for the local administration of nanoplatforms, when appropriate, thereby limiting systemic exposure and increasing the therapeutic concentration at the tumor site [6]. This unique feature makes OSCC particularly suitable for the development and evaluation of advanced nanotheranostic systems. Moreover, the integration of the concept of multifunctionality has also led to the development of systems that can be applied in the field of phototherapy. In particular, photodynamic therapy (PDT) and photothermal therapy (PTT) represent two minimally invasive approaches that exploit light irradiation to selectively induce tumor cell death, in which light stimulation can be used to control drug release or to simultaneously activate multiple therapeutic mechanisms, promoting synergistic strategies that combine phototherapy, chemotherapy, immunotherapy, or catalytic effects [7].

In light of these considerations, this review provides an updated overview of the main multifunctional theranostic nanoplatforms developed for the treatment of OSCC. We will further analyze the various classes of nanomaterials under investigation, including lipid, polymeric, inorganic, carbon-based, biomimetic, and hybrid-based nanoplatforms, highlighting their targeting strategies, integrated imaging modalities, and the potential of therapeutic combinations based on PDT and PTT. The review further explores how the integration of nanomedicine, diagnostic imaging, and phototherapy may contribute to the development of more selective, personalized, and image-guided therapeutic strategies, while addressing the major challenges that currently limit their clinical translation in OSCC.

## 2. Oral Squamous Cell Carcinoma: Clinical Challenges and Unmet Needs

OSCC is the most common malignancy of the oral cavity, accounting for approximately 90% of all oral cancers. Globally, it is considered the sixth most prevalent cancer, with more than 350,000 new cases and nearly 300,000 deaths reported each year. Despite significant advances in both diagnosis and treatment over the past decades, the prognosis for patients with OSCC remains poor, with a 5-year survival rate of only 40–50%. The high mortality associated with OSCC is largely attributable to its pronounced invasive and metastatic potential, which promotes local disease progression and dissemination to adjacent head and neck structures, placing it within the broader spectrum of head and neck squamous cell carcinomas (HNSCCs) [8]. Paradoxically, although the oral cavity is readily accessible for clinical examinations, a considerable proportion of patients are still diagnosed at advanced stages. This is often due to the nonspecific appearance of early lesions or their misinterpretation during routine clinical evaluation, ultimately delaying appropriate treatment. OSCC is a multifactorial disease, arising from the interplay of several environmental and biological factors. Among the best-established risk factors that contribute to the malignant transformation of the oral epithelium are tobacco use, excessive alcohol consumption, human papillomavirus (HPV) infection, immune dysregulation, and a variety of additional genetic and environmental factors [9]. Diagnosis primarily relies on histopathological examination of biopsy specimens, supported by imaging techniques that are essential for assessing tumor extension and disease staging. Tumor stage remains the main criterion guiding therapeutic decision-making. Surgery is still considered the standard treatment for resectable lesions; however, extensive tissue resection often results in permanent functional and aesthetic impairments, substantially affecting patients’ quality of life. Radiotherapy represents the most widely adopted non-surgical approach, but it can be associated with significant damage to surrounding healthy tissues and long-term adverse effects. Likewise, chemotherapy, which is commonly administered in combination with surgery and/or radiotherapy, is limited by poor tumor selectivity, systemic toxicity, and the frequent development of drug resistance. Over the last decade, targeted therapies and immunotherapeutic approaches have expanded the therapeutic landscape for OSCC [10]. Currently, the main agents approved by the U.S. Food and Drug Administration (FDA) include cetuximab, an anti-epidermal growth factor receptor (EGFR) monoclonal antibody, and programmed cell death protein 1 (PD-1) immune checkpoint inhibitors such as nivolumab. Although these treatments have represented an important step forward compared with conventional therapies, their overall clinical benefits remain modest. In particular, anti-EGFR therapy has provided only limited improvements in patient survival over standard chemotherapy, whereas anti-PD-1 immunotherapy is effective only in a subset of patients and may be associated with immune-related adverse events [11].

Taken together, these limitations underscore the urgent need for more effective therapeutic strategies capable of improving treatment selectivity while reducing systemic toxicity. At the same time, there is a growing demand for approaches that enable earlier diagnosis and real-time monitoring of therapeutic response. In this regard, the anatomical accessibility of the oral cavity offers a unique opportunity for the development of precision medicine strategies based on multifunctional theranostic nanoplatforms.

## 3. Nanotheranostics and Phototherapy in OSCC

### 3.1. Nanotheranostics: Towards Image-Guided Precision Medicine

During last years, current therapeutic strategies have led to growing interest in the development of approaches designed to improve treatment efficacy as well as selectivity toward the tumor site. In this regard, nanomedicine has progressively played a central role in cancer research, offering the opportunity to design multifunctional nanosystems capable not only of simply delivering drugs but, going beyond the mere concept of drug delivery systems, of integrating various therapeutic and diagnostic functions within a single platform, named as theranostic [12]. Indeed, thanks to their size, generally less than 100 nm, and their chemical and physical versatility, these nanosystems are engineered to improve drug stability, prolong their circulation time, and promote preferential accumulation at the tumor site through active targeting mechanisms achieved by functionalization with selective targeting agents. Specifically, next-generation nanoplatforms are capable of integrating, in addition to the therapeutic payload, multimodal imaging (fluorescence, photoacoustic, magnetic resonance, computed tomography, and ultrasound), along with controlled release mechanisms triggered by internal or external stimuli, depending on the conditions of the tumor microenvironment (TME), or by external stimuli such as near-infrared (NIR) irradiation or magnetic fields [13]. These multifunctional nanoplatforms allow for the observation of nanoparticle biodistribution, thereby monitoring their accumulation at the tumor site and tracking the response to treatment over time using various imaging techniques. In this way, this approach can be considered part of the field of personalized medicine, in which therapeutic treatment is no longer viewed as a static process but is dynamically adapted in response to the progression of the disease [14].

Thanks to the high versatility of these nanosystems, the literature provides numerous examples of theranostic applications using a wide variety of materials, including those of lipid, polymeric, and inorganic origin, as well as carbon-based materials, biomimetic systems, and hybrid platforms. In particular, in the case of OSCC, the anatomical location makes the oral cavity easily accessible both for the local administration of nanosystems and for the direct monitoring of the therapeutic response. Consequently, the integration of imaging and therapy represents one of the most promising avenues for developing approaches that are increasingly selective, personalized, and guided in real time by the progression of the disease.

In this review, the term theranostic specifically refers to nanoplatforms that integrate therapeutic functions with diagnostic imaging capabilities. However, the role of imaging may vary considerably among these systems. In some platforms, imaging is primarily used to assess nanoparticle biodistribution and tumor accumulation, whereas more advanced image-guided approaches exploit imaging information to guide treatment and monitor therapeutic response over time. Selected nanoplatforms lacking an imaging component are also discussed throughout the review because of their relevance to OSCC treatment, including active targeting, drug or gene delivery, and phototherapeutic strategies. Although these systems are not considered theranostic in the strict sense, they provide valuable functional approaches that may contribute to the development of more integrated nanotheranostic platforms.

### 3.2. Phototherapy as a Cornerstone of Multifunctional Theranostic Nanoplatforms

As noted above, the latest-generation multifunctional nanosystems are often characterized by their ability to respond to external stimuli, such as light radiation. In this regard, phototherapy has emerged in recent years as a promising strategy for cancer treatment, thanks to its excellent properties, including minimal invasiveness, high efficacy, selectivity, and low toxicity [15].

Phototherapy essentially comprises PDT and PTT. In the first case, light is used to generate reactive oxygen species (ROS) from a photosensitizer (PS), which, upon irradiation with light at a specific wavelength, generates ROS capable of inducing cellular death due to redox imbalance and immune responses [16]. During PTT, on the other hand, the photothermal agent, following light stimulation at a specific wavelength, undergoes oscillatory relaxation, releasing non-radiative energy, in the form of heat. This heating can result in mild-to-moderate hyperthermia when temperatures reach approximately 41–43 °C, leading to cell death by apoptosis. When temperatures exceed 47 °C, however, tissue necrosis, coagulation, and carbonization are induced [17].

Certainly, in both PDT and PTT, the external light source is a key factor. In this regard, NIR light is the most desirable option in terms of both its ability to penetrate tissues and its lower absorption and scattering in biological tissues [18]. Furthermore, NIR-induced anticancer phototherapy allows for greater selectivity toward cancer cells, since these are more sensitive to phototherapy than healthy cells. Therefore, a good phototherapeutic agent should have the following characteristics: (i) good responsiveness to NIR, sufficient to induce the production of ROS (PDT) or heat (PTT); (ii) good biodegradability or bioeliminability to prevent accumulation that could lead to toxic effects; (iii) good selectivity toward cancer cells following light stimulation; (iv) and the ability to be easily functionalized with therapeutic agents or targeting agents [19].

Therefore, the objective of this review is to focus on the application of multifunctional theranostic nanosystems in the field of OSCC, with particular emphasis on the potential synergistic effects of anticancer treatment combined with phototherapeutic applications such as PDT or PTT.

## 4. Classification of Smart Theranostic Nanoplatforms

### 4.1. Organic Nanoplatforms

Organic theranostic nanoparticles are among the most widely studied nanocarriers for the diagnosis and treatment of cancer, thanks to their excellent biocompatibility, biodegradability and versatile physicochemical properties. They are generally composed of natural or synthetic biomacromolecules, including lipids, polymers, proteins and carbohydrates, which enable the development of multifunctional systems that simultaneously deliver therapeutic agents and imaging probes [20]. Their high colloidal stability, efficient drug loading, good dispersibility in aqueous media and ease of surface functionalization make them particularly suitable for targeted and controlled drug delivery applications [21]. Furthermore, the marked stability of organic nanoparticles in biological fluids, combined with their ability to effectively retain and protect encapsulated therapeutic payloads, makes them particularly suitable as systems responsive to external stimuli, notably light and heat [22]. It is interesting that many organic nanomaterials respond to NIR light due to the presence of extended π-conjugated structures [23]. Overall, these characteristics make organic nanoparticles extremely promising platforms for numerous biomedical applications, including targeted drug delivery, bioimaging, biosensing and diagnostics (Table 1) [20].

Among organic nanoplatforms, liposomes represent one of the most widely investigated lipid-based nanoplatforms to develop advanced multifunctional theranostic nanosystems for the treatment of cancer.

In this context, Wei et al. [24] developed a multifunctional liposomal theranostic nanoplatform for a trimodal anti-tumor treatment of squamous cell carcinoma of the tongue (CAL27 and FaDu models). The liposomal formulation has been designed to simultaneously deliver evodiamine (EVO) and indocyanine green (ICG), integrating therapeutic and diagnostic functions into a single system. Thanks to this combination, the nanosystem has been used as a platform for bimodal imaging using fluorescence imaging (FI) and positron emission tomography/computed tomography (PET/CT). A particularly interesting aspect concerns EVO, which has been used as a chelating agent for the positron-emitting radionuclide ^68^Ga, thereby providing an effective contrast agent for PET/CT. In addition to this diagnostic role, EVO has also demonstrated peroxidase-like activity, promoting the conversion of hydrogen peroxide (H_2_O_2_) present in the TME into ROS and thereby contributing to the effect of chemodynamic therapy (CDT). Following irradiation with an 808 nm NIR laser (808 nm, 100 mW/cm^2^, 1 min), the applied light triggered the release of EVO from the liposomal nanoplatform, while simultaneously activating ICG-mediated PDT. The combination of these mechanisms led to a marked synergistic cytotoxic effect, resulting from the combined action of chemotherapy, CDT and PDT [24].

In addition to conventional liposomes, other lipid-based nanoplatforms, such as porphysomes (PorNPs), have recently attracted considerable interest in theranostic applications. Unlike conventional liposomes, which generally require the encapsulation of imaging agents and therapeutic payloads, porphysomes consist of phospholipid–porphyrin conjugates that spontaneously self-assemble to form liposome-like structures with both diagnostic and therapeutic properties. These nanoparticles exhibit fluorescence in the NIR region and can be used in PDT following irradiation at 671 nm [25,26]. A distinctive feature of PorNPs is their compact structural organization, which, in the assembled state, significantly reduces both fluorescent emission and the production of singlet oxygen. This behavior limits the unwanted activation of the photosensitizer in healthy tissues. Once inside the cells, however, the nanoparticles tend to disassemble, releasing the porphyrin–lipid conjugates and thereby regaining their fluorescent and photodynamic properties. Thanks to this intracellular activation process, PorNPs exhibit greater selectivity towards tumor tissue, enhancing the efficacy of PDT whilst reducing the risk of damage to surrounding tissues [27].

The theranostic potential of PorNPs has been further demonstrated in preclinical models of OSCC. In this regard, Sahovaler et al. [26] investigated the use of PorNPs for the selective treatment of OSCC using fluorescence-guided PDT. The selectivity of this approach stems from the greater tendency of these nanoparticles to accumulate in tumor tissue compared with the surrounding healthy tissues. This behavior was confirmed in vivo using three different preclinical models—in mice and rabbits—which demonstrated a significantly higher retention of PorNPs in tumor lesions within the oral cavity. Following intracellular uptake, the nanoparticles underwent disassembly, resulting in the restoration of the porphyrin’s photoactive properties: the fluorescence intensity at 675 nm (100 mW/cm^2^) increased by approximately 100-fold, whilst singlet oxygen production was approximately four times higher. Following light irradiation, this reactivation resulted in a marked induction of apoptosis and the consequent death of tumor cells, demonstrating the potential of PorNPs as a platform for selective and effective PDT in the treatment of OSCC [26]. These findings suggest that porphysomes represent a promising evolution of conventional liposomal systems, combining tumor-selective activation with integrated imaging and photodynamic therapy.

Beyond lipid-based nanoplatforms, polymeric nanoparticles (PNPs) represent another important class of organic nanomaterials. Their properties can vary significantly depending on the polymer used; however, their low toxicity is a key characteristic that makes them particularly attractive as drug and gene delivery systems. Furthermore, PNPs can be engineered for active targeting through surface functionalization with specific ligands [20]. Their high loading capacity also allows for the incorporation of PSs, enabling their use in phototherapeutic strategies [28].

A representative example was reported by Wang J. and colleagues who developed a multifunctional chitosan/tripolyphosphate (CS–TPP)-based nanoplatform to combine gene therapy and photodynamic therapy in the treatment of OSCC. The nanoparticles were produced via ionic cross-linking and co-loaded with 5-aminolevulinic acid (5-ALA) and a short hairpin RNA targeting methylene tetrahydrofolate dehydrogenase 1-like (MTHFD1L), which plays a key role in OSCC cell proliferation, resulting in an average nanosystem size of approximately 145 nm. The silencing of MTHFD1L was designed to selectively induce apoptosis in neoplastic cells, whilst minimizing effects on healthy tissues. Meanwhile, the release of 5-ALA, a precursor of protoporphyrin IX, increased the intracellular photosensitization of CAL27 and SCC25 cells, thereby enhancing the efficacy of PDT following 635 nm laser irradiation (10 J/cm^2^). The combination of these two therapeutic strategies, tested both in vitro models and in vivo in orthotopic OSCC mouse models, led to a marked inhibition of cell proliferation and the induction of apoptosis [29].

In another study, Wang Y. and colleagues developed a system of targeted polymeric nanoparticles with the aim of increasing therapeutic selectivity against OSCC. The nanoparticles, produced by nanoprecipitation and loaded with DOX, were subsequently functionalized with the HN-1 peptide, thereby yielding the targeted HN-1 nanoparticles (HNPDs). HN-1 is known for its ability to effectively cross cell membranes and for its tendency to be preferentially internalized by HNSCC cells. In line with these characteristics, HNPDs exhibited significantly higher cytotoxicity in OSCC cell lines (CAL27 and SCC25) compared with non-targeted nanoparticles (PDs). In contrast, no significant differences were observed between HNPDs and PDs in HepG2 hepatoma cells, suggesting that the greater accumulation of HNPDs in OSCC cells is attributable to a specific internalization mechanism mediated by HN-1 [30].

In addition to conventional lipid- and polymer-based systems, advanced nanoplatforms have attracted increasing attention for theranostic applications. Among these, self-assembled nanoparticles are of particular interest due to their relatively simple preparation, which is generally based on an ultrasound-assisted mixing process. These conditions promote the spontaneous organization of the components into nanometer-scale structures through non-covalent interactions. This approach yields highly stable nanoplatforms, characterized by efficient drug delivery properties, as well as high biocompatibility and biodegradability [31].

Xu and colleagues developed a multifunctional system of carrier-free, self-assembled nanoparticles for PDT of OSCC guided by bimodal FI and MRI (magnetic resonance imaging). The nanoparticles were prepared by incubating, under continuous stirring, the chlorin e6 (Ce6) PS—which is responsible for both fluorescence emission and photodynamic activity following NIR laser irradiation (660 nm, 50 mW/cm^2^, 5 min)—with sorafenib in an aqueous solution containing ferric chloride (FeCl_3_). The resulting system (Sor–Ce6 NPs) was formed through π–π stacking interactions, hydrogen bonding and coordination with Fe^3+^ ions. Notably, the exogenous Fe^3+^ incorporated into the Sor–Ce6 NPs helped to alleviate tumor hypoxia via Fenton reactions, improving photodynamic performance whilst simultaneously increasing ROS production and reducing glutathione peroxidase 4 (GPX4) expression, thereby triggering cell death via ferroptosis. In vivo anti-tumor efficacy studies conducted on nude mice bearing CAL27 xenografts confirmed the ability of Sor–Ce6 NPs to effectively inhibit tumor growth. From an imaging perspective, the combined presence of Ce6 and Fe^3+^ ions have endowed the nanoplatform with theranostic properties, enabling bimodal fluorescent imaging and T1-weighted MRI [32].

Metal–organic frameworks (MOFs) represent another important class of organic nanosystems. MOFs are self-assembled systems consisting of metal ions and organic ligands linked by coordination bonds, and they have attracted considerable interest in the field of drug delivery due to their high surface area, structural stability, tunable pore sizes, and wide range of functionalization possibilities [33]. Among the various MOFs studied, mesoporous iron (III) trimesate (MIL-100) is one of the most widely used in PDT applications. In this structure, trimesic acid acts as an organic ligand, coordinating Fe^3+^ ions. The high porosity and good biocompatibility of MIL-100 make it particularly suitable for drug loading and release [34].

In this context, Dhawan and collaborators have developed hybrid nanoplatforms consisting of a core formed by FeAu alloy nanoparticles coated with MIL-100(Fe)-type MOFs, resulting in the FeAu@MOF system. DOX was encapsulated within the MOF structure, resulting in a superparamagnetic theranostic nanoplatform designed for MRI-guided magneto-chemo-thermotherapy of OSCC. The study also systematically assessed the influence of the number of MOF layers (5 or 10) surrounding the FeAu core. An increase in the number of MIL-100(Fe) layers resulted in greater cytocompatibility in murine fibroblast cells (L929), as well as an increase in DOX loading capacity and its controlled release in the presence of an alternating magnetic field. Overall, FeAu@MOF nanostructures demonstrated significant potential both as MRI contrast agents and as therapeutic platforms, inducing approximately 90% apoptosis in human OSCC cells (HSC-3) after HFIW (700–1000 KHz) for 10 min of magnetic hyperthermia and resulting in an approximately 30-fold reduction in tumor volume in a vivo murine xenograft model [35].

**Figure 1:**
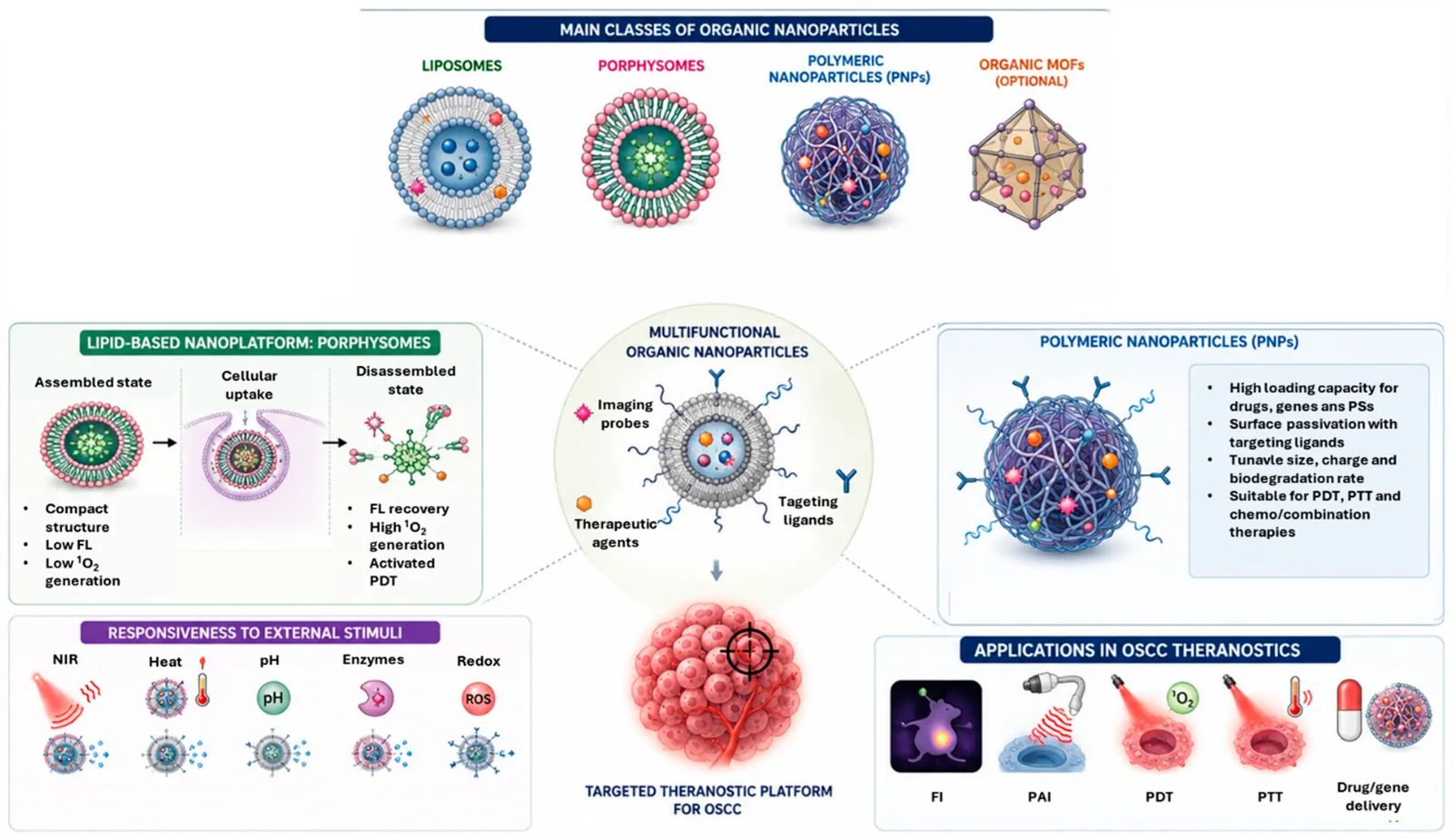
Schematic overview of the main classes of organic theranostic nanoplatforms investigated for OSCC, highlighting their structural features, functionalization strategies, imaging capabilities, and therapeutic applications, including chemotherapy, PDT and PTT.

Overall, organic nanoplatform offer highly versatile theranostic systems thanks to their excellent biocompatibility, structural flexibility and ability to integrate multiple imaging and therapeutic modalities. Lipid-based systems, polymeric nanoparticles, self-assembled nanostructures and MOFs have all shown promising preclinical results for the diagnosis and treatment of OSCC (Figure 1). However, several challenges remain to be addressed before their clinical translation can be achieved. Further studies are needed to validate the efficacy of emerging platforms such as porphyrins in clinical settings, whilst polymeric nanoparticles still present limitations related to the variability in degradation kinetics and drug release profiles. Furthermore, the long-term biodistribution, biosafety and pharmacokinetic behavior of many organic nanoplatforms have yet to be fully elucidated. Addressing these issues will be essential for the successful clinical implementation of organic theranostic nanosystems in OSCC.

**Table 1:** Summary of organic nanoplatforms investigated for theranostic applications in OSCC.

Nanoplatform	Imaging	Therapeutic Strategy	Targeting	OSCC Model/Administration	Main Findings/Limitations
Liposomal nanoplatform [24]	FI + PET/CT	Chemotherapy + CDT + PDT (808 nm, 100 mW/cm^2^, 1 min)	-	CAL-27, FaDu; orthotopic CAL-27 tongue xenograft; i.y.	Synergistic trimodal therapy with bimodal imaging; long-term in vivo safety not assessed
PorNPs nanoparticles [26]	FI	PDT 675 nm (100 mW/cm^2^)	-	Two murine xenograft models and one orthotopic rabbit model; i.v. (tail vein in mice; marginal ear vein in rabbits).	Tumor-selective photoactivation and fluorescence-guided PDT; clinical validation remains lacking
CS-TPP nanoparticles [29,30]	-	Gene therapy + PDT (635 nm, 10 J/cm^2^)	-	CAL-27, SCC-25; orthotopic SCC-25 mouse model	Enhanced PDT through MTHFD1L silencing; no integrated imaging capability
HNPD nanoparticles [30]	-	Chemotherapy	HN-1 peptide	CAL-27; SCC-25; subcutaneous SCC-25 xenograft; i.v.	HN-1 enhanced OSCC-selective uptake and cytotoxicity; therapeutic validation remains limited
Sor–Ce6 self-assembled nanoparticles [32]	FI + MRI	PDT (660 nm, 50 mW/cm^2^, 5 min) + Ferroptosis/CDT	-	CAL-27; subcutaneous CAL-27 xenograft; i.v. (tail vein)	Effective tumor inhibition with bimodal imaging
FeAu@MOF [35]	MRI	Magneto-chemo-thermal therapy (HFIW, 10 min)	-	HSC-3; HSC-3 xenograft; i.v.	Strong magneto-chemo-thermal efficacy with MRI capability; complex hybrid formulation may hinder translation

### 4.2. Inorganic Nanoplatforms

**Figure 2:**
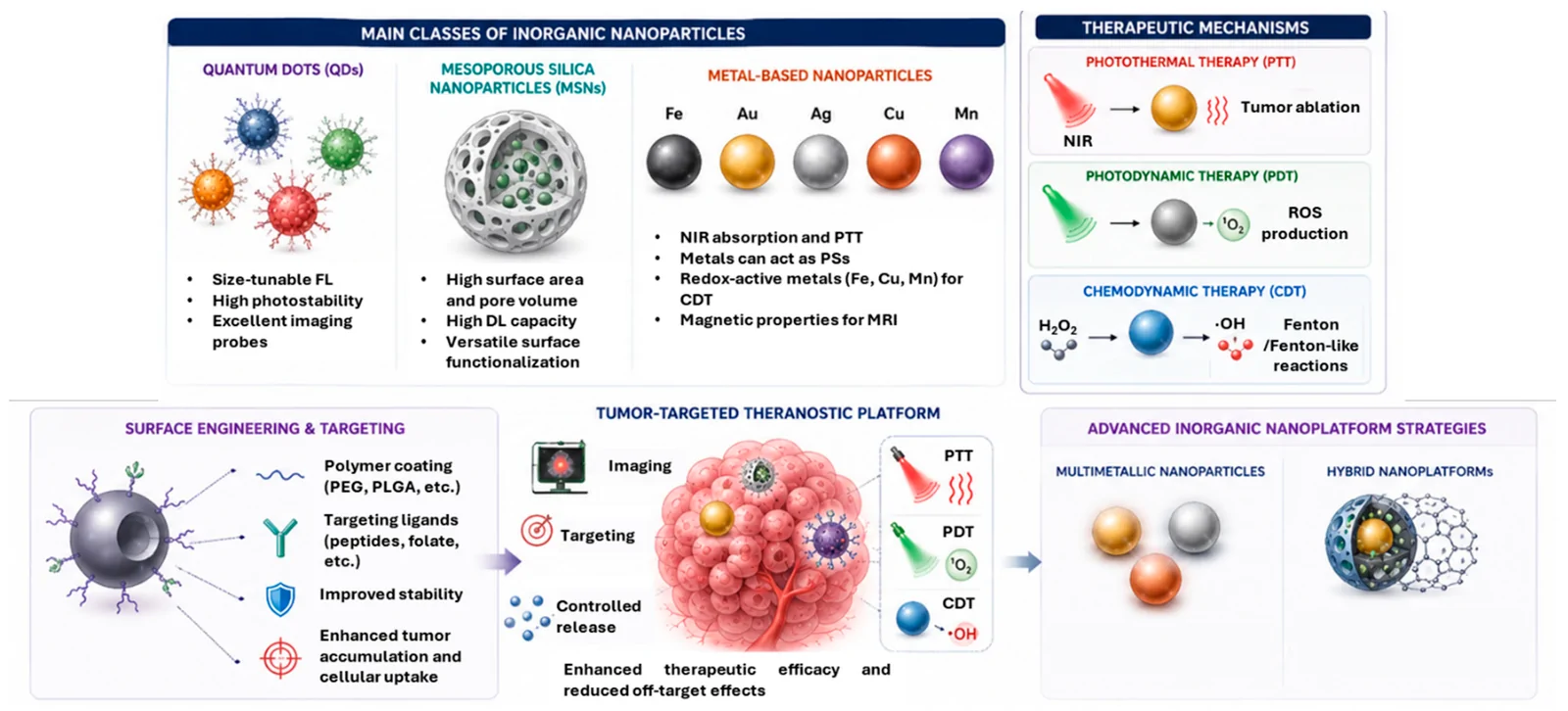
Representative inorganic nanomaterials for OSCC theranostics, including metal-based nanoparticles, MSNs, QDs, and hybrid nanoplatforms, with their principal imaging and therapeutic functions.

Inorganic nanoparticles are classified as a broad class of materials characterized by highly organized three-dimensional structures and intrinsic optical, thermal, magnetic and catalytic properties [20]. The category of inorganic nanoparticles includes quantum dots (QDs), mesoporous silica nanoparticles (MSNs) and metal-based nanoparticles, which are composed of iron (Fe), gold (Au), silver (Ag), copper (Cu) or manganese (Mn), which currently represent one of the most extensively studied categories for biomedical and theranostic applications (Figure 2, Table 2). Indeed, the presence of metals confers particularly interesting characteristics on these systems, such as high absorption in the NIR region, the ability to efficiently convert light into heat and, in some cases, the potential to act directly as PSs for PDT. Furthermore, redox-active metals such as iron, copper and manganese can catalyze Fenton or Fenton-like reactions, promoting the generation of ROS from endogenous hydrogen peroxide (H_2_O_2_) and contributing to the elimination of tumor cells via chemodynamic therapy (CDT) [36]. Compared with organic nanosystems, inorganic nanoparticles offer the advantage of being easily modified during synthesis through the introduction of metallic elements, enabling the simultaneous integration of diagnostic and therapeutic functionalities without the need for subsequent chemical modifications [37]. However, their application in biological contexts can be limited by issues of colloidal stability and biocompatibility that is not always optimal. For this reason, surface coating with polymers is a frequently adopted strategy to improve their stability and circulation in physiological environment and, at the same time, to promote selective accumulation at the tumor site through the conjugation of targeting ligands [38].

Of all the inorganic nanoplatforms proposed for the treatment of OSCC, metal-based nanoparticles are undoubtedly the most widely reported in the literature [39]. Depending on their composition, they can be categorized into systems based on silver, iron, copper, gold or manganese-based nanoparticles, as well as more complex hybrid nanoplatforms that combine different metallic components or integrate different types of materials within the same nanostructure.

A particularly noteworthy example is provided by the Ag_2_S QDs developed by Xiong and colleagues. These nanosystems, obtained via hydrothermal synthesis, were targeted with an anti-podoplanin (PDPN) monoclonal antibody, a biomarker associated with partial epithelial–mesenchymal transition (pEMT), a cellular state involved in OSCC invasion and metastasis. Thanks to this functionalization, Ag_2_S QDs were able to selectively recognize SCC-7 cells and, at the same time, provide fluorescent images in the NIR-II region. Upon irradiation with 808 nm laser (1.0 W/cm^2^), the targeted Ag_2_S QDs exhibited a rapid rise in temperature to approximately 60 °C within 5 min, inducing an effective photoimmunotherapy (PIT) effect. It is interesting to note that irradiation of non-targeted Ag_2_S QDs did not produce significant cytotoxic effects, whilst a marked reduction in cell viability was observed only in the presence of PDPN-mediated targeting, highlighting the crucial role of active tumor recognition in achieving therapeutic efficacy. Through PIT, in vivo studies on SCC-7 xenograft models revealed that the treatment was able not only to slow tumor growth but also to remodeling the immunosuppressive microenvironment, reducing the infiltration of regulatory T cells and tumor-associated macrophages (TAMs) and promoting the activation of dendritic cells. Furthermore, combination of the targeted Ag_2_S QDs with anti-PD-1 therapy further increased the infiltration of CD4^+^ and CD8^+^ T lymphocytes, suggesting that this strategy may potentiate antitumor immunity and help overcome resistance to immunotherapy [40].

Alongside multifunctional theranostic systems, simpler metallic nanoparticles have also been investigated for the treatment of OSCC. For example, Zhang and colleagues developed Prussian Blue nanoparticles (PB NPs) stabilized with polyvinylpyrrolidone (PVP) and investigated their ability to modulate the TME. Unlike most theranostic nanoplatforms, PB NPs were not designed for imaging or phototherapy applications, but to promote the repolarization TAMs from the pro-tumor M2 phenotype to the anti-tumor M1 phenotype. In vitro studies revealed a reduction in the CD206 marker and an increase in the CD86 marker, accompanied by modulation of the expression of various inflammatory cytokines. Furthermore, PB NPs disrupted the pro-tumor dialogue between TAMs and OSCC cells, inhibiting tumor progression in the SCC-7 xenograft models whilst maintaining good cytocompatibility with human oral keratinocytes (HOKs) [41]. Similarly, metallic gold nanoparticles (AuNPs) have been investigated as photothermal agents for the treatment of OSCC. Following laser irradiation at 807 nm (50 mW/cm^2^), AuNP-mediated PTT induced significant apoptosis in SCC-27 cells, as demonstrated by increased expression of cytochrome c and caspase-8, associated with the modulation of c-Myc. Although this study did not exploit imaging capabilities or additional therapeutic modalities, it provided initial proof of concept regarding the use of gold-based nanomaterials as photothermal agents for the treatment of OSCC [42].

Although conventional metal nanoparticles have demonstrated some therapeutic benefits in the treatment of OSCC, their potential for application is often limited by the absence of integrated diagnostic functions, reduced targeting capabilities and reliance on a single therapeutic mechanism. Consequently, research has progressively shifted towards the development of multifunctional nanoplatforms capable of integrating different metallic elements or hybrid architectures, combining imaging properties, catalytic activity, and responsiveness to specific stimuli within a single system, with the aim of improving therapeutic efficacy and expanding theranostic potential. In this context, Liu and colleagues developed hollow, mesoporous Fe-Cu sulphide-based nanospheres coated with hyaluronic acid (HA) and further functionalized with the mitochondrial ligand TPP. The nanocatalyst was subsequently loaded with BAY-876, a glucose transporter (GLUT1) inhibitor, resulting in a multifunctional theranostic platform capable of exerting a dual targeting mechanism via both the enhanced permeability and retention (EPR) effect and CD44-mediated endocytosis, thanks to the presence of HA.

In addition, the intrinsic Fe^3+^ content endowed the nanosystem with MRI contrast capability. The nanoplatform was designed to amplify cuproptosis, an emerging form of regulated cell death driven by mitochondrial copper accumulation and specific metabolic vulnerabilities. Following cellular internalization, the acidic TME induced the release of Fe^2+^, Cu^+^, H_2_S and BAY-876, triggering a self-amplifying cascade characterized by oxidative stress, mitochondrial dysfunction, ATP depletion and glutathione consumption. Specifically, H_2_S promoted the intracellular accumulation of H_2_O_2_, fueling Fe- and Cu-mediated Fenton and Fenton-like reactions and leading to massive production of hydroxyl radicals. Concurrently, BAY-876 inhibited GLUT1-mediated glucose uptake and glycolysis, causing a severe bioenergetic crisis that promoted the mitochondrial accumulation of copper and the activation of cuproptosis. The efficacy of HA-mediated targeting was confirmed by ICP-OES analysis, which revealed significantly higher intracellular levels of Fe and Cu compared with untreated controls (2.4 ± 0.2 and 3.0 ± 0.3 pg/cell versus 0.2 ± 0.1 and 0.3 ± 0.1 pg/cell, respectively). Furthermore, the metabolic inhibition induced by BAY-876 led to a marked reduction in GLUT1 expression and glycolytic activity, with intracellular ATP levels reduced to approximately 16% of those in untreated CAL-27 cells. Overall, these synergistic effects led to a significant inhibition of tumor growth in CAL-27 murine xenograft models following intravenous administration [43].

Collectively, these findings highlight the potential of multicomponent inorganic nanoplatforms to simultaneously exploit catalytic activity, metabolic modulation, targeted delivery, and diagnostic imaging within a single nanosystem. Further evidence supporting the versatility of multimetallic theranostic platform was reported by Li et al. [44], who developed TiO_2_@PDA-MnDopa nanocomposites by coating TiO_2_ nanoparticles with polydopamine (PDA) and Mn-Dopa chelates through a horseradish peroxidase (HRP)-mediated surface polymerization process. The high catalytic efficiency of HRP enabled the simultaneous deposition of PDA and Mn-Dopa onto the TiO_2_ surface, yielding a multifunctional nanosystem specifically designed to integrate MRI with synergistic PTT and PDT. Despite TiO_2_ is a well-established PS, its clinical application is hindered by its predominant responsiveness to ultraviolet light, which limits its effectiveness in deep-seated tumors. To address this limitation, PDA was introduced to extend light absorption into the NIR region and simultaneously enhance photothermal conversion efficiency. As a result, the nanocomposite exploited the photocatalytic properties of TiO_2_ for PDT, while PDA acted as the photothermal component, enabling a synergistic PTT/PDT effect under 808 nm laser irradiation (2.0 W/cm^2^). Notably, this combined phototherapeutic approach reduced HSC-3 cell viability to less than 5% following laser exposure. Furthermore, incorporation of Mn-Dopa endowed the nanosystem with efficient T1-weighted MRI contrast capability. Particularly, the longitudinal and transverse relaxivities (r_1_ and r_2_) increased by 5.22-fold compared with free Mn-Dopa, demonstrating the beneficial effect of chelate immobilization on the TiO_2_ surface. In this sense, in vivo MRI studies in CAL-27 tumor-bearing mice confirmed efficient tumor accumulation after intravenous administration and enabled the identification of the optimal irradiation window, approximately 1 h post-injection. The significant suppression of tumor growth observed in vivo demonstrated the superior efficacy of the combined PTT/PDT strategy compared with either modality alone, highlighting the advantages of integrating diagnostic imaging and synergistic phototherapy within a single nanoplatform [44].

In addition to multimetallic nanosystems, in which the integration of different metallic elements allows for the creation of multifunctional systems with complementary properties, hybrid nanoplatforms also emerged as a promising strategy for OSCC theranostics. These nanosystems are generally obtained by combining different types of nanomaterials within a single architecture, with the aim of integrating their respective physicochemical and biological characteristics. This strategy allows for the development of more advanced platforms, capable of combining diagnostic features, targeting capabilities, and multiple therapeutic approaches within a single system, significantly expanding its theranostic potential for the treatment of OSCC. In that sense, Zeng et colleagues developed a hybrid core–shell nanoplatform consisting of a core made of gold nanorods (AuNRs) coated with a mesoporous silica layer. The surface of the nanostructure was subsequently functionalized with HA by an amidation reaction, giving the system the ability to selectively recognize OSCC cells overexpressing the CD44 receptor. Finally, DOX was loaded into the mesoporous shell through electrostatic interactions. The resulting nanoplatform (DOX-AuNRs@mSiO2-HA) performed a dual function: on the one hand, it acts as a contrast agent for photoacoustic imaging (PAI), thanks to the presence of AuNRs, on the other, it is able to convert NIR irradiation into heat, allowing the application PTT while promoting triggered drug release. Following CD44 receptor-mediated internalization, enzymatic degradation of the HA coating by hyaluronidases promoted DOX release, an effect further amplified by laser irradiation at 808 nm (2.0 W cm^−2^, 5 min). Under physiological conditions only approximately 24% of the encapsulated DOX was released over 24 h, while exposure to NIR light increased drug release to over 60%, highlighting the system’s marked responsiveness to light stimuli. PAI was further employed to monitor the biodistribution of DOX-AuNRs@mSiO_2_-HA, revealing maximal tumor accumulation approximately 8 h after intravenous administration, which was subsequently identified as the optimal time point for laser irradiation. The application of chemo-photothermal therapy resulted in effective tumor ablation in mice bearing CAL-27 xenografts, while maintaining good cytocompatibility with HOK [45].

Furthermore, it is interesting to note that in another study, conducted by Zhou et al. [46], the anti-tumor effect of the co-delivery of two anti-cancer drugs, docetaxel and cisplatin, for the treatment of OSCC was investigated. The carrier selected was MnO_2_-based mesoporous nanoshells, subsequently stabilized with polyethylene glycol (PEG) and simultaneously co-loaded with both drugs (TP), thereby yielding the H-MnO_2_-PEG/TP system. In addition to acting as a drug delivery system, MnO_2_ played a fundamental role in conferring theranostic properties to the nanoplatform. In fact, the nanoshells proved to be unstable when incubated in an acidic environment, due to their degradation with the release of Mn^2+^ ions, a phenomenon that ensured both the precise and controlled release of the drugs at the tumor site and the activation of MRI contrast properties. This aspect has enabled the development of a system highly responsive to the TME and capable of acting as a T1-MRI contrast agent specific to neoplastic tissue; following administration in CAL-27 xenograft models, a marked increase in the T1 signal was observed in the tumor region compared to muscle tissue, attributed to the conversion of MnO_2_ to Mn^2+^. Concurrently, the degradation of MnO_2_ has made it possible to modulate the TME through the simultaneous release of Mn^2+^, which triggered the catalytic decomposition of H_2_O_2_, producing oxygen. This mechanism helped to alleviate the hypoxic conditions typical of the tumor, as confirmed by a reduction in the expression of hypoxia-inducible factor 1-alpha (HIF-1α), a factor closely associated with the progression and metastasis of OSCC [46].

**Table 2:** Summary of inorganic nanoplatforms investigated for theranostic applications in OSCC.

Nanoplatform	Imaging	Therapeutic Strategy	Targeting	OSCC Model/Administration	Main Findings/Limitations
αPDPN-Ag_2_S QDs [40]	NIR-II FI	PIT (808 nm, 1.0 W/cm^2^) + immunomodulation	Anti-PDPN antibody	SCC-7 cells; subcutaneous SCC-7 xenograft; i.v.	Selective NIR-II imaging and PIT and enhanced antitumor immunity with anti-PD-1
Prussian Blue nanoparticles [41]	-	Immuno-modulation (TAM repolarization)	-	HSC-3/HN-6/CAL-27 cells; SCC-7 ± RAW 264.7 xenografts; peritumoral injection	Effective TAM M2-to-M1 repolarization; no integrated imaging capability
Au NPs [42]	-	PTT (807 nm, 50 mW/cm^2^)	-	SCC-27 cells; in vitro only	Effective photothermal apoptosis; in vitro proof-of-concept only
B@FCSHMN@HA-TPP [43]	MRI	Cuproptosis + CDT + metabolic therapy	HA/CD44 and mitochondrial TPP	CAL-27 cells; subcutaneous CAL-27 xenograft; i.v. (tail vein)	Effective tumor inhibition through targeted cuproptosis and metabolic modulation; complex multicomponent design
TiO_2_@PDA-MnDopa [44]	*T1*-weighted MRI	Synergistic PTT/PDT (808 nm, 2.0 W/cm^2^)	-	HSC-3 cells; HSC-3 xenograft; i.v. (tail vein)	MRI-guided synergistic PTT/PDT
DOX-AuNRs@ mSiO_2_-HA [45]	PAI	Chemo-PTT (808 nm, 2.0 W/cm^2^, 5 min)	HA/CD44	CAL-27 cells; subcutaneous CAL-27 xenograft; i.v. (tail vein)	PAI-guided chemo-PTT with enhanced DOX release
H-MnO_2_-PEG/TP [46]	*T1*-weighted MRI	Chemotherapy + TME modulation	-	CAL-27/SCC-27 cells; CAL-27 xenograft; i.v.	TME-responsive drug release and MRI activation

### 4.3. Carbon-Based Nanomaterials

**Figure 3:**
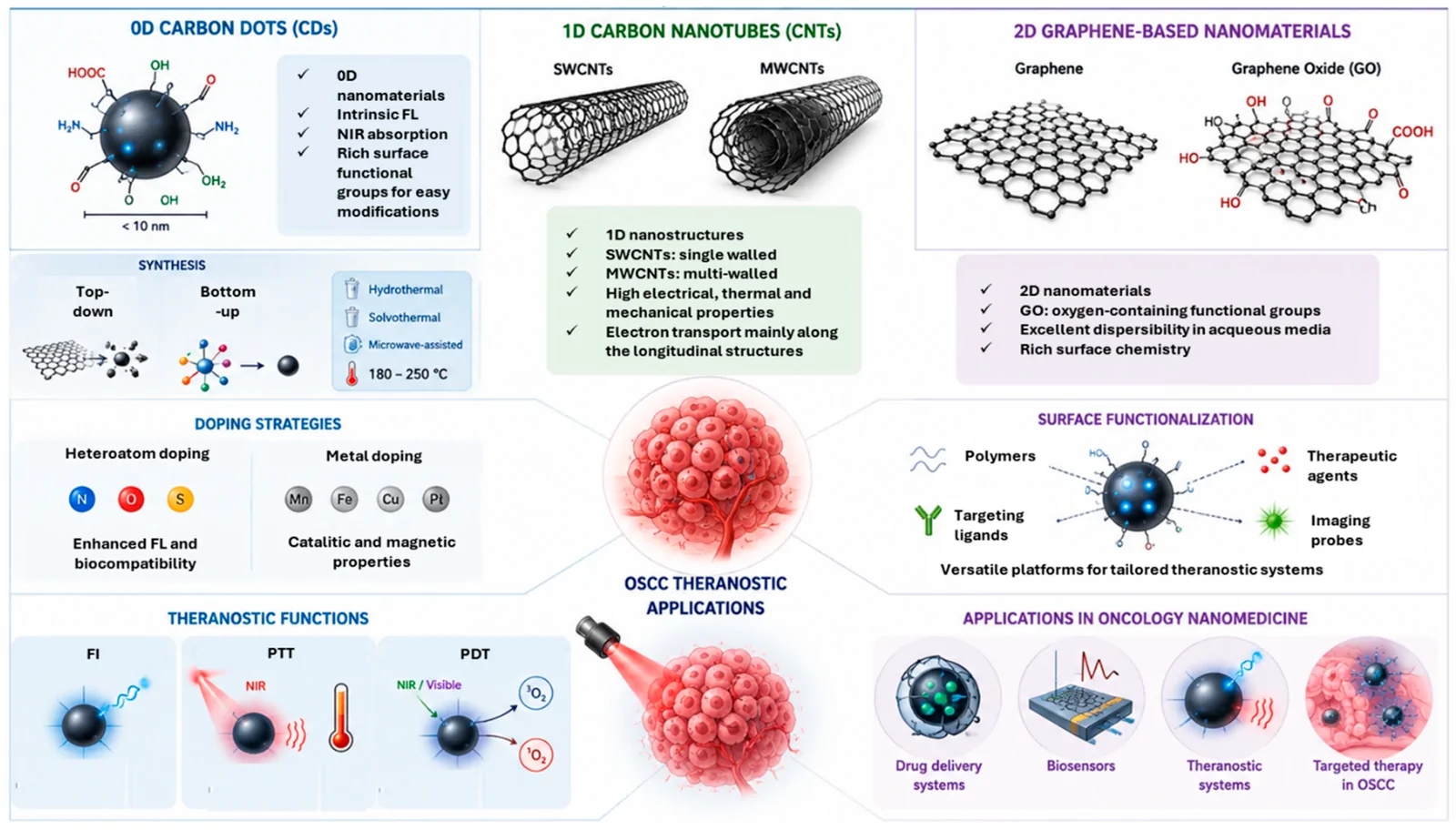
Overview of carbon-based nanomaterials investigated for OSCC theranostics, including CDs (0D), CNTs (1D), and graphene-based nanomaterials (2D), highlighting their main structural features and applications in imaging, drug delivery, PTT and PDT.

Along with inorganic nanoplatforms and polymeric nanosystems, carbon-based nanomaterials have attracted increasing interest in cancer nanomedicine due to their combination of unique properties such as chemical stability, tunable optical properties, efficient electrical and heat conduction, and brilliant photoluminescence [47]. These properties have allowed their application in a wide variety of fields, including drug delivery systems, biosensors, bioimaging and theranostic systems. This category of nanomaterials includes a wide range of nanostructures, including carbon dots (CDs), graphene oxide (GO), reduced graphene oxide, carbon nanotubes (CNTs), and other nanostructured forms of carbon, each characterized by specific physicochemical properties that influence its biological behavior and potential therapeutic applications (Figure 3, Table 3) [48].

Surely, within this macro-category, CDs have emerged as particularly versatile nanomaterials for biomedical and theranostic applications. These nanomaterials can be synthesized through either top-down or bottom-up approaches, which involve, respectively, the size reduction in larger carbonaceous structures or the carbonization of molecular precursors by hydrothermal, solvothermal, or microwave-assisted treatments, typically performed at temperatures ranging from 180 to 250 °C [49]. Furthermore, CDs are zero-dimensional (0D) nanomaterials, generally characterized by sizes below 10 nm and a surface rich in functional groups, providing numerous opportunities for surface passivation and functionalization with polymers, targeting ligands, and therapeutic agents, thereby facilitating the development of tailored theranostic platforms [50]. Regarding their imaging properties, a distinctive feature of CDs is their intrinsic fluorescence, which has prompted extensive investigation of these nanomaterials as optical imaging probes. In addition, depending on their composition, synthetic precursors, doping, and surface states, selected CDs can exhibit absorption extending into the NIR region and promote photothermal conversion or ROS generation, enabling their application in PTT and PDT, respectively. In this regard, the rational selection of precursors plays a crucial role, as it allows the engineering of CDs capable of simultaneously acting as photosensitizers and fluorescent imaging agents, thereby integrating diagnostic and therapeutic functionalities within a single nanoplatform [51].

Interestingly, as previously discussed for inorganic nanoparticles, CDs can also be doped with heteroatoms to tailor their physicochemical and biological properties. In most cases, synthetic precursors are selected to introduce oxygen-, nitrogen- or sulphur- containing species into the carbon framework, which generally enhance the photoluminescence performance of the resulting nanomaterials [52]. However, doping strategies can also involve the incorporation of metallic elements, providing additional functionalities beyond fluorescence.

In this context, Zhang et al. [53] developed manganese-doped carbon dots (Mn-CDs) with an average diameter of approximately 2.7 nm for the treatment of OSCC. Besides preserving the intrinsic optical properties of CDs, manganese incorporation endowed the nanosystem with catalase (CAT)-like activity, enabling the decomposition of H_2_O_2_ and the modulation of the TME. Under 635 nm laser irradiation (300 mW/cm^2^, 5 min), Mn-CDs efficiently generated singlet oxygen (^1^O_2_), demonstrating their potential as PS for PDT. To evaluate their catalytic and photodynamic performance under tumor-mimicking conditions, OSCC-9 cells were incubated with Mn-CDs in a mildly acidic medium (pH 6.5) supplemented with H_2_O_2_ (50 μM). Under these conditions, the combined treatment dramatically reduced cell viability to only 3.58%, highlighting the synergistic contribution of CAT-like activity and PDT in enhancing antitumor efficacy [53].

Additional evidence supporting the potential of CDs as photodynamic agents for OSCC treatment has been provided by studies exploiting their intrinsic photosensitizing properties. For instance, sulfur-doped carbon dots (S-CDs) have been investigated as fluorescent nanoprobes and PDT agents in UM1 oral cancer cells. Thanks to their intrinsic red fluorescence emission, S-CDs enabled in vitro FI while simultaneously acting as PS under LED irradiation at 420 nm. Their photodynamic efficacy was compared with that of 5-ALA, a clinically established precursor of protoporphyrin IX. Under identical experimental conditions, S-CD-mediated PDT resulted in higher ROS generation and a more pronounced reduction in cell viability, suggesting a superior photo-oxidative activity compared with 5-ALA at the same concentration [54]. Even in another study, it was explored the theranostic potential of CDs endowed with intrinsic fluorescence and photodynamic activity in CAL-27 and UM1 cells. Upon irradiation in the UV range (360–370 nm, 100 mW/cm^2^ for 3 min), the CDs promoted intracellular ROS generation and consequent tumor cell death, while simultaneously providing FI capabilities [55]. Further interesting work related to CDs-based nanosystems for OSCC treatment was reported by Das et al. [56], who developed a hybrid nanostructured system made of nitrogen-rich mesoporous carbon nanospheres embedding ultrasmall nitrogen-doped carbon quantum dots (NCQD-HCS). The mesoporous carbon matrix was synthesized from poly(pyrrole-co-aniline) in the presence of Triton X-100 as a structure-directing agent, while the resulting material was subsequently carbonized under an inert atmosphere. After evaluating different carbonization temperatures, 800 °C was identified as the optimal condition, yielding a nanoplatform of approximately 65–70 nm and characterized by a relatively high fluorescence quantum yield (14.6% at 340 nm) together with remarkable photothermal conversion efficiency (54.6%) under 980 nm laser irradiation (1.4 W cm^2^). NCQD-HCS combined the fluorescence properties of the embedded QDs with the photothermal performance of the carbon nanosphere matrix. Upon NIR irradiation, NCQD-HCS induced a pronounced temperature increase and exhibited significant thermal ablation activity against FaDu oral cancer cells [56]. Taken together, these studies demonstrate the considerable potential of CDs for OSCC theranostics, particularly owing to their intrinsic fluorescence and their ability to generate heat or ROS upon light irradiation. However, in many cases, the reported systems have been evaluated mainly in vitro, while their performance in more complex biological settings remains largely unexplored. Moreover, a number of these platforms rely on UV or visible-light activation, which inevitably limits tissue penetration and may reduce their applicability in the treatment of deeper tumor lesions. Future efforts should therefore focus on the development of more advanced CD-based nanoplatforms capable of combining efficient NIR responsiveness with targeted delivery and multimodal imaging functionalities, ultimately improving their translational potential for OSCC management.

Moving from 0D carbon-based nanomaterials to one-dimensional (1D) ones, CNTs represent one of the most extensively investigated 1D carbon-based nanostructures. CNTs can be described as single- or multi-layered graphene sheets, referred to as single-walled carbon nanotubes (SWCNTs) and multi-walled carbon nanotubes, respectively, that are rolled into cylindrical nanostructures. They are classified as 1D materials because their morphology extends predominantly along a single axis, resulting in electron transport that is mainly confined to the longitudinal direction. This unique structural organization gives rise to remarkable electrical, thermal, and mechanical properties, which are strongly influenced by the number of graphene layers constituting the nanotube architecture [57]. Among the most interesting applications of CNTs in OSCC, Wang and co-workers developed a CD44-targeted hybrid nanocomplex by conjugating ICG to HA nanoparticles and subsequently incorporating them SWCNTs, generating the ICG-HANP/SWCNTs (IHANPT) nanosystem. Within this multifunctional architecture, HA served as an active targeting ligand toward CD44-overexpressing tumor cells, while SWCNTs and ICG played complementary roles in both phototherapy and imaging. Specifically, SWCNTs were responsible for the photothermal effect, whereas ICG acted both as a PS for PDT and as a contrast agent for PAI. In vitro studies performed on SCC7 cells revealed a pronounced synergistic interaction between PTT and PDT. Upon 808 nm laser irradiation (0.3 W/cm^2^), IHANPT exhibited a markedly higher photothermal conversion capability than control formulations containing either free ICG or HA nanoparticles alone, rapidly increasing the temperature from approximately 25 °C to 55 °C within less than four minutes. Interestingly, the authors identified this temperature range as an optimal therapeutic window, as it allowed the preservation of both the photodynamic activity and the chemical stability of ICG, thereby maximizing the combined PTT/PDT effect. From a diagnostic perspective, PAI was exploited to monitor, in real time, the biodistribution and tumor accumulation of the nanosystem. Following intravenous administration in SCC7 xenograft-bearing mice, the photoacoustic signal associated with IHANPT progressively increased within the tumor region, confirming efficient accumulation mediated by CD44-targeted delivery. In contrast, administration of free ICG generated only negligible photoacoustic signals, highlighting the limited ability of the PS to selectively accumulate within tumor tissue in the absence of a targeted nanocarrier [58].

The last class of carbon-based nanomaterials investigated for anticancer applications of OSCC is represented by graphene. Graphene consists of a single layer of carbon atoms arranged in a two-dimensional honeycomb lattice. Its atomically thin structure, with a thickness of less than 1 nm, provides an exceptionally high surface-to-volume ratio, making it highly reactive and chemically versatile. These features have attracted considerable interest in biomedical applications, particularly in drug delivery and cancer theranostics [59]. Besides pristine graphene, the family of two-dimensional (2D) carbon nanomaterials also includes GO, an oxidized derivative characterized by the presence of abundant oxygen-containing functional groups on its surface. Owing to its improved dispersibility in aqueous media and its rich surface chemistry, GO has emerged as one of the most widely explored graphene-based materials for the development of multifunctional nanoplatforms in cancer diagnosis and therapy [60]. Among graphene-based nanomaterials, Li et al. [61] developed a multifunctional nanographene oxide (NGO)-based theranostic platform for targeted chemo-photothermal therapy of OSCC. The nanosystem was obtained by first conjugating NGO with PEG through EDC/NHS-mediated coupling; moreover, PEG can be employed as a additional material for graphene. Beyond its role in improving the physicochemical properties of the nanosystem, PEG has been reported to activate macrophages and may contribute to enhancing antitumor immune responses. Subsequently, a fibroblast activation protein (FAP)-targeting peptide labeled with a fluorescent probe was grafted onto the nanoparticle surface, obtaining the targeted nanocarrier NPF. Finally, DOX was loaded through π–π stacking interactions between the aromatic rings of the drug and the NGO structure, generating the final formulation, NPF@DOX. The rationale behind this design relies on the selective targeting of FAP, a type II transmembrane glycoprotein highly expressed by cancer-associated fibroblasts (CAFs) within the OSCC’s TME, that plays a key role in tumor growth, invasion, and metastatic progression. The NGO platform served as a photothermal agent under 808 nm laser irradiation (1.0 W/cm^2^, 5 min), enabling the combination of chemo-photothermal therapy. Furthermore, the nanocarrier exhibited a dual pH- and temperature-responsive drug release behavior. Interestingly, under physiological conditions (pH 7.4), only about 34% of the loaded DOX was released over 72 h, whereas drug release increased to approximately 67% at pH 5.5, mimicking the acidic intracellular compartments of tumor cells. Lastly, in vivo studies confirmed efficient tumor accumulation after intravenous administration and demonstrated significant inhibition of tumor growth following the combined chemo-photothermal treatment [61]. In a similar work conducted by the same research group, GO nanosystem was co-targeted with HA and the HN-1 peptide for the controlled release of DOX in the tumor site (DOX@GO-HA-HN-1). The dual targeting strategy was designed with the aim of exploiting the complementary targeting capabilities of HA and HN-1, thereby enhancing nanoparticle accumulation and cellular internalization in OSCC cells compared with conventional single-targeting approaches. Indeed, confocal microscopy revealed a markedly stronger intracellular fluorescence signal in CAL-27 cells treated with DOX@GO-HA-HN-1 compared with the corresponding single-targeted formulations, indicating more efficient cellular uptake. These observations were further confirmed by flow cytometry analyses, which demonstrated that the HA/HN-1 combination promoted significantly higher nanoparticle internalization than either targeting ligand alone. In contrast, uptake by HOK remained minimal, suggesting good selectivity toward tumor cells. Interestingly, nanoparticle internalization was further enhanced under acidic conditions (pH 5.0) combined with laser irradiation at 808 nm (1.0 W/cm^2^, 5 min), aligned with the intracellular trafficking and release mechanisms of the nanosystem [62].

Lastly, Chen et al. [63] explored amino-functionalized graphene oxide (AGO) as a photothermal agent for OSCC treatment. Compared with GO, AGO carries a positive surface charge, which is generally considered advantageous because it can favor stronger interactions with the negatively charged membranes of cancer cells and, in turn, improve cellular uptake and tumor penetration. AGO was simply obtained by first sonicating and then heating ethylenediamine with the GO dispersion. Moreover, this surface modification also improved the photothermal response: under 808 nm laser irradiation (6 W/cm^2^, 6 min), AGO quickly heated up, reaching temperatures above 57 °C within just a few minutes, whereas unmodified GO showed almost no temperature increase under the same conditions. This stronger photothermal effect translated into effective tumor ablation both in HSC-3 cells and in xenograft-bearing mice after intratumoral injection followed by NIR irradiation [63].

**Table 3:** Summary of carbon-based nanomaterials investigated for theranostic applications in OSCC.

Nanoplatform	Imaging	Therapeutic Strategy	Targeting	OSCC Model/Administration	Main Findings/Limitations
Mn-doped CDs [53]	-	PDT + CAT-like activity (635 nm, 300 mW cm^−2^, 5 min)	-	OSCC-9 cells; in vitro only	Efficient ^1^O_2_ generation and CAT-like activity; in vitro validation only
S-doped CDs [54]	FI	PDT (420 nm)	-	UM1 cells; in vitro only	Higher ROS generation than 5-ALA; in vitro validation only
CDs [55]	FI	PDT (360–370 nm, 100 mW/cm^2^ for 3 min)	-	CAL-27/UM1 cells; in vitro only	Combined FI and PDT; UV excitation limits therapeutic applicability
NCQD-HCS [56]	FI	PTT (980 nm, 1.4 W/cm^2^)	-	FaDu cells; in vitro only	High fluorescence quantum yield and photothermal efficiency; in vitro validation only
IHANPT (ICG-HANP/SWCNTs) [58]	PAI	Synergistic PTT/PDT (808 nm, 0.3 W/cm^2^)	HA/CD44	SCC7 cells; SCC7 xenograft; i.v. (tail vein)	Synergistic PDT/PTT with PA-guided tumor accumulation
FAP-targeted PEGylated NGO (NPF@DOX) [61]	FI (Cy5.5)	Chemo-PTT (808 nm, 1.0 W/cm^2^, 5 min)	FAP-targeting peptide	CAL-27 cells; subcutaneous CAL-27 xenograft; i.v. (tail vein)	FAP-targeted, pH/NIR-responsive DOX delivery with effective tumor inhibition
Dual-targeted GO (GHHD) [62]	FI (Cy5.5)	PTT (808 nm, 1.0 W/cm^2^, 5 min)	HA (CD44) + HN-1 peptide	CAL-27 cells; subcutaneous CAL-27 xenograft; i.v. (tail vein)	Dual targeting enhanced selective cellular uptake
Amino-modified Graphene Oxide (AGO) [63]	-	PTT (808 nm, 6 W/cm^2^, 6 min)	-	HSC-3 cells; HSC-3 xenograft; i.t.	Enhanced photothermal heating and tumor ablation

### 4.4. Hybrid Hydrogels

Among the hybrid platforms recently investigated for the treatment of OSCC, injectable hydrogels also deserve special attention. These are three-dimensional systems consisting of polymeric networks capable of incorporating nanoparticles, drugs, or other bioactive molecules and releasing them in a controlled manner at the site of administration. Unlike conventional nanosystems administered systemically, hydrogels offer the advantage of being injected directly into the tumor lesion, forming a local therapeutic deposit that prolongs the retention of therapeutic agents, limits their systemic distribution, and reduces potential side effects. This approach is particularly promising for the treatment of OSCC, as anatomical accessibility of oral tumors facilitates intratumoral or peritumoral administration. Furthermore, the integration of functional nanoparticles into the hydrogel matrix allows the advantages of nanomedicine to be combined with those of biomaterials, resulting in hybrid systems capable of integrating controlled release, responsiveness to external stimuli, such as NIR irradiation, and, in some cases, modulation of the TME. Thanks to these properties, hydrogels represent a promising strategy not only for improving local therapeutic efficacy but also for prolonging the retention of therapeutic agents at the tumor site, reducing systemic exposure, and potentially promoting more durable antitumor responses (Table 4) [64].

In this context, Fu and colleagues developed an injectable hybrid hydrogel composed of CS and hydroxyethylcellulose (HEC), used as a matrix to incorporate benzothiadiazole-based organic photothermal nanoparticles. The incorporation of these nanoparticles into the polymeric network was based on the goal of limiting the dissipation of heat generated during NIR irradiation, promoting its confinement within the tumor site and thereby improving the efficiency of PTT. Thanks to the nanoparticles’ high photothermal conversion efficiency, exceeding 40%, the system was able to reach temperatures of approximately 75 °C after only 3 min of irradiation at 808 nm (0.5 W/cm^2^, 10 min), resulting in a marked photothermal ablation effect.

**Figure 4:**
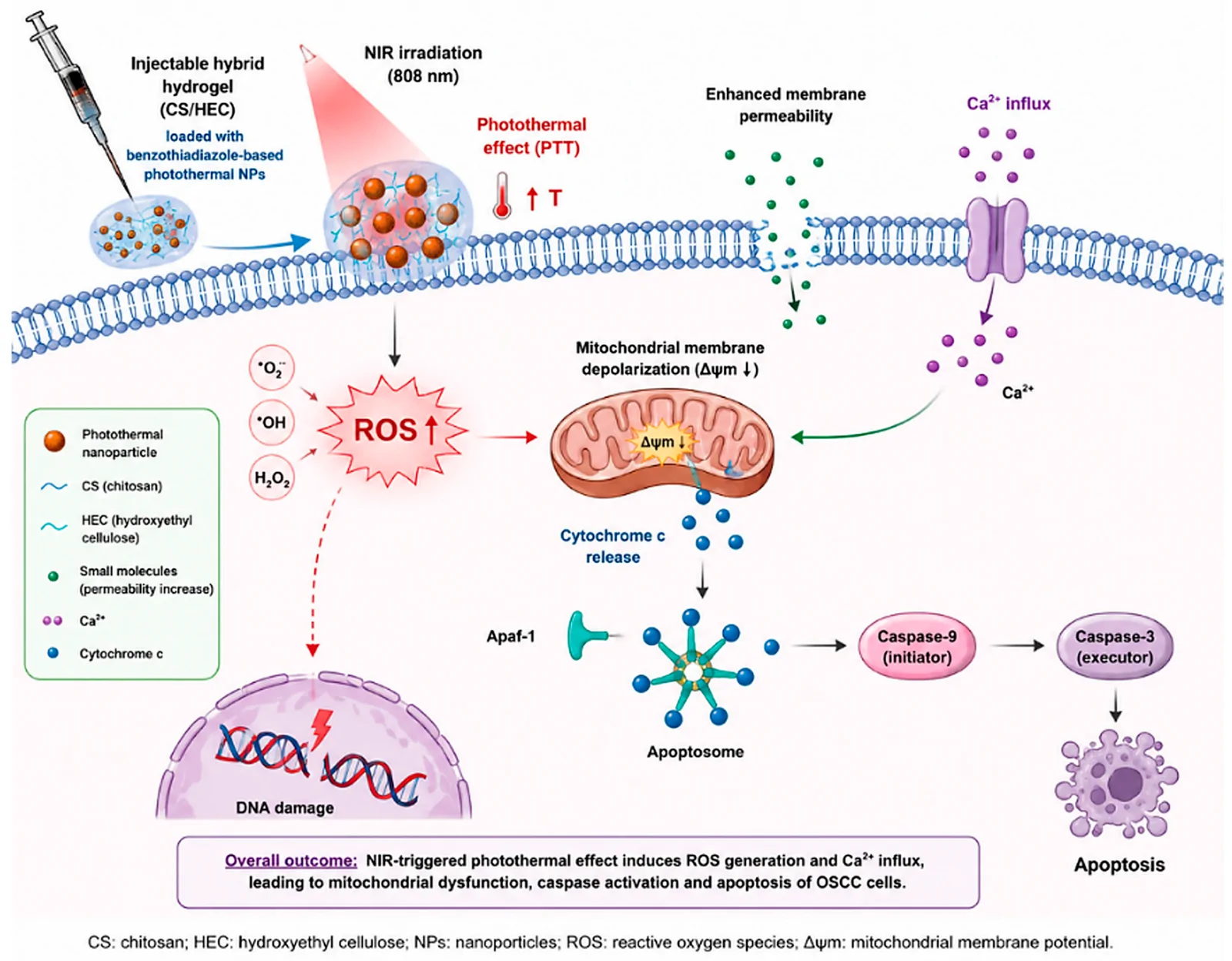
Proposed mechanism of photothermal therapy-induced apoptosis mediated by the injectable hybrid hydrogel. NIR irradiation promotes photothermal heating, ROS generation, and Ca^2+^ influx, leading to mitochondrial dysfunction, cytochrome *c* release, caspase activation, and ultimately OSCC cell apoptosis. Schematic representation based on the mechanism proposed by [65].

The authors also investigated the biological mechanisms responsible for the antitumor activity. In particular, local heating led to a significant increase in intracellular production of ROS, accompanied by an increase in plasma membrane permeability and a consequent influx of Ca^2+^. This event triggered the depolarization of the mitochondrial membrane potential, compromising the correct cell function and inducing apoptosis in tumor cells (Figure 4). These results were confirmed in both the CAL-27 and SCC-25 cell lines, while reduced cytotoxicity was observed in HOK and normal squamous epithelial cells (NOK). Therapeutic efficacy was subsequently validated in an orthotopic murine model of OSCC established by inoculating CAL-27 cells into the tongue, in which intratumoral administration of the hydrogel, followed by NIR irradiation, resulted in marked inhibition of tumor growth [65].

**Figure 5:**
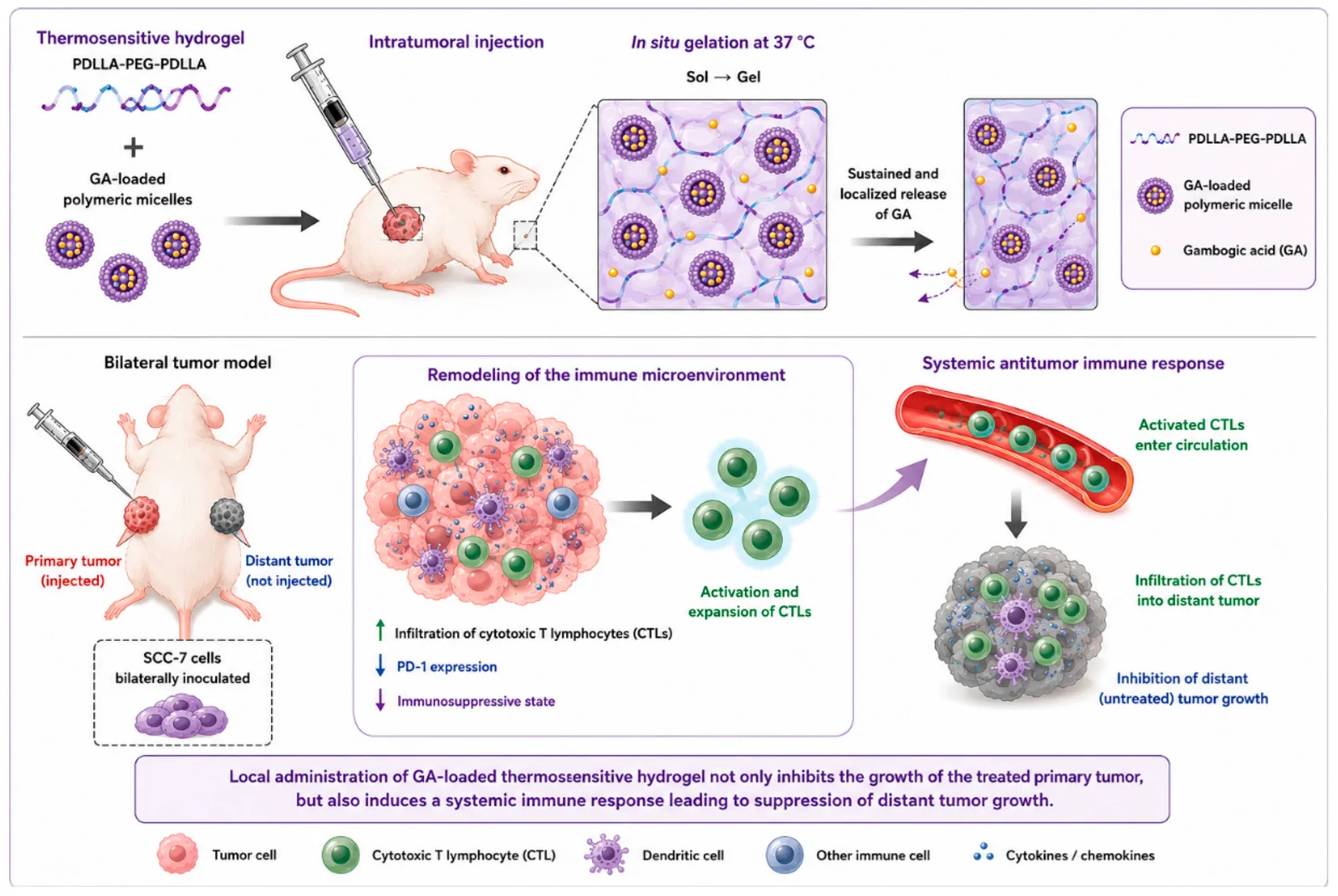
Antitumor effects of the GA-loaded thermosensitive hydrogel. Local GA release promotes primary tumor inhibition and immune activation, leading to systemic suppression of distant tumor growth. Schematic representation based on [66].

Another example is the thermosensitive hydrogel developed by Chen et al. [66], prepared from a thermosensitive material based on a triblock copolymer consisting of poly(D,L-lactide) (PDLLA) end blocks and a central PEG segment (PDLLA-PEG-PDLLA) and loaded with polymeric micelles containing gambogic acid (GA), a natural compound with promising antitumor properties but one that has yet to be fully explored in the treatment of OSCC. Following intratumoral injection, the system underwent in situ gelation at 37 °C, enabling prolonged and localized drug release. In addition to causing a marked inhibition of primary tumor growth, the treatment was found to remodel the immune microenvironment, promoting the infiltration of cytotoxic T lymphocytes and reducing PD-1 expression, thereby attenuating the tumor’s immunosuppressive state. Of particular interest was the experimental model used by the authors, in which SCC-7 cells were bilaterally inoculated into mice, while the GA-loaded hydrogel was administered to only one of the two tumors (primary tumor). Surprisingly, in addition to the marked inhibition of primary tumor growth in the treated tumor, a significant reduction in the growth of the untreated contralateral tumor (distant tumor) was also observed, suggesting that the local therapy could trigger a systemic antitumor response mediated by activation of the immune system. This result highlights the potential of injectable hydrogels not only as effective local drug delivery systems but also as immunomodulatory platforms capable of extending the therapeutic effects of locoregional treatment to distant tumor sites (Figure 5) [66].

### 4.5. Biomimetic Nanoparticles

**Figure 6:**
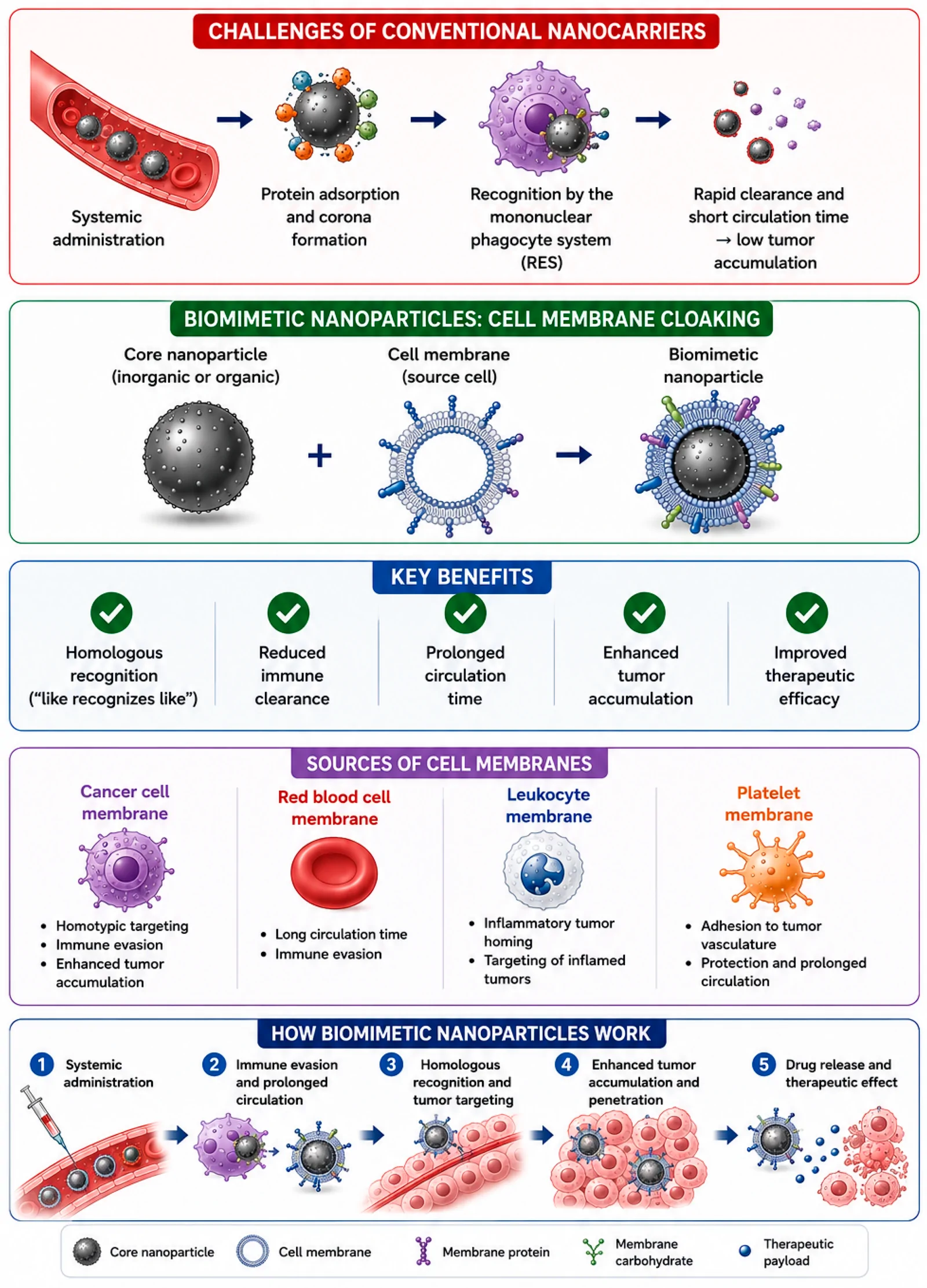
Overview of biomimetic nanoplatforms for OSCC therapy. Biomimetic strategies exploit cell membranes to enhance tumor targeting, immune evasion, and therapeutic efficacy.

While injectable hydrogels provide an effective strategy for improving local drug retention, systemically administered nanocarriers face different biological barriers that can substantially limit their therapeutic performance. Among these, rapid recognition and clearance by the immune system represent major challenges. Following systemic administration, nanoparticles are often rapidly coated with plasma proteins, leading to the formation of a protein corona on their surface. This process can promote their recognition by the mononuclear phagocyte system (reticuloendothelial), ultimately reducing blood circulation time and limiting drug accumulation at the tumor site [67]. To overcome these limitations, increasing attention has recently been directed toward biomimetic nanotherapeutics, an emerging class of nanosystems inspired by the concept that “like recognizes like”. In these platforms, inorganic or organic nanoparticles can be coated with cell membranes derived from cancer cells, red blood cells, leukocytes, or platelets. By preserving key membrane proteins and carbohydrates from the source cells, these biomimetic coatings enable homologous recognition of target tissues, reduce immune clearance, and prolong systemic circulation, ultimately improving tumor accumulation and therapeutic efficacy (Table 5) [68]. The main biomimetic strategies investigated for OSCC treatment, and their associated therapeutic advantages are summarized in Figure 6.

This strategy has also been explored for OSCC treatment. For example, H. Li et al. [69] developed DOX encapsulated poly(lactic-co-glycolic acid) (PLGA) nanoparticles (DOX-NPs) for chemotherapy and subsequently coated them with a cancer cell membrane. In order to obtain a stimulus-responsive system, the membrane was functionalized with DSPE-PEG_2000_ conjugated to ICG, yielding a biomimetic nanoplatform specifically designed for combined chemo-photothermal therapy. The cancer cell membrane conferred homologous targeting capability, promoting preferential accumulation within OSCC tissue while reducing immune recognition, obtaining the final system known as lip-CC@DOXNPs. At the same time, ICG acted as the photothermal agent under NIR irradiation. Interestingly, laser exposure not only generated localized hyperthermia, at 808 nm (1.5 W/cm^2^, 5 min), but also accelerated DOX release through disruption of the membrane coating, thus enhancing drug availability directly within the tumor. An interesting aspect of this study was the comparison between two different in vivo models: a conventional subcutaneous xenograft model, generated by inoculating HSC-3 cells into the flank of mice, and a more clinically relevant orthotopic model, in which tumor cells were implanted into the tongue. While complete tumor ablation was achieved in the xenograft model following treatment with lip-CC@DOXNPs combined with NIR irradiation, the orthotopic model provided a more realistic evaluation of therapeutic performance because it better reproduced the native oral TME. In this setting, free DOX and conventional DOX-NPs achieved tumor growth inhibition rates of approximately 18% and 41%, respectively, whereas coating the nanoparticles with the cancer cell membrane increased inhibition to 52%, highlighting the contribution of homologous targeting. Remarkably, combining the biomimetic nanoplatform with NIR irradiation further increased tumor growth inhibition to nearly 91%, demonstrating the clear therapeutic advantage of integrating homologous targeting with synergistic chemo-photothermal therapy [69].

Beyond cancer cell membrane-coated nanoparticles, biomimicry can also be achieved by exploiting living immune cells as active drug delivery vehicles. In this context Xu et al. [70], engineered neutrophils as living drug delivery vehicles for targeted photo-chemotherapy of OSCC. The authors isolated neutrophils from murine bone marrow and loaded them with a multifunctional nanocomplex (RA/Ce6) composed of Ce6-loaded liposomes physically associated with positively charged arginine–glycine–aspartic acid (RGD) peptides, yielding photoactive neutrophils (PANs). This design was intended to take advantage of the intrinsic tumor-homing ability of neutrophils, which naturally migrate toward inflammatory sites, allowing selective accumulation within the TME while avoiding rapid immune clearance. In addition, the RA/Ce6 payload provided a second level of targeting through RGD-mediated recognition of αvβ3 integrins, which are highly expressed by OSCC cells. Besides facilitating cellular uptake, RGD promoted mitochondrial localization and induced mitochondrial membrane depolarization, making tumor cells more susceptible to ROS-mediated damage during PDT.

After intravenous administration, the engineered PAN actively migrated toward the inflammatory TME and subsequently released their payload in response to inflammatory stimuli. Under 660 nm laser irradiation (100 mW/cm^2^, 5 min), the simultaneous production of ROS induced by Ce6, together with the inflammatory response triggered by PDT further recruited additional neutrophils, creating a positive feedback mechanism that enhanced tumor accumulation after repeated treatments. Indeed, the chemotactic behavior was also corroborated by in vivo studies, where repeated cycles of laser irradiation and intravenous injection progressively increased PAN retention within the tumor, confirming that PDT-induced inflammation further amplified neutrophil recruitment [70].

A further biomimetic approach involves the use of naturally derived extracellular vesicles as drug carriers. M. Li and colleagues developed a nanoplatform based on exosomes derived from OSCC cells (SCC-7), used as delivery systems for ICG and gefitinib, an EGFR tyrosine kinase inhibitor, frequently overexpressed in OSCC. The resulting system (IG@EXOs) was designed to combine an EGFR- targeted molecular therapy with synergistic phototherapy (PTT/PDT) under 785 nm laser irradiation (0.5 W cm^2^, 5 min). The use of exosomes as carriers represents a particularly interesting approach, as these extracellular vesicles retain the biological characteristics of their parental cells, promoting a homologous targeting mechanism against the tumor. Following tumor accumulation, laser irradiation simultaneously induced the production of heat and ROS by the ICG, resulting in a combined PTT/PDT effect. In addition to directly promoting tumor cells apoptosis, light stimulation also facilitated a controlled gefitinib release and its subsequent translocation into the cytoplasm, thereby amplifying the inhibition of the EGFR signaling pathway and inducing the synergistic phototherapy–molecular targeted therapy (PMTT). In mouse models of OSCC, IG@EXOs demonstrated an efficient tumor accumulation, resulting in significant regression of the tumor mass along with marked inhibition of lymph node metastasis, highlighting the potential of exosomes as biomimetic platforms for the integration of homologous targeting, molecular therapy, and phototherapy [71].

Despite the extremely promising results obtained with biomimetic systems based on cell membranes, one of the main limitations of the current literature lies in the scarcity of truly theranostic platforms. In most cases, in fact, these nanosystems have been designed primarily to improve tumor targeting and therapeutic efficacy, without integrating true imaging capabilities capable of monitoring, in real time, the biodistribution of the nanocarrier, treatment response, or tumor progression. The development of biomimetic nanoplatforms capable of combining homogeneous targeting, multimodal therapy, and diagnostic imaging therefore represents one of the main future challenges for facilitating their translation into precision OSCC treatment.

## 5. Comparative Assessment of Nanoplatforms for OSCC Theranostics

The comparative analysis of the different nanoplatforms investigated for OSCC reveals substantial differences in their physicochemical properties, which in turn influence three aspects of particular relevance to their potential application: biological fate and clearance, intrinsic imaging capability, and versatility as drug delivery systems (Table 6).

Organic nanoparticles generally provide a favorable balance between biocompatibility and biodegradability, largely because they are commonly composed of lipids, including phospholipids (i.e., DSPC, DOPE, and DPPE) or biocompatible polymers (i.e., PEG, PMeOx, and biodegradable polyesters) [72,73]. Depending on their composition, these materials can undergo chemical or enzymatic degradation, facilitating the subsequent elimination of their degradation products through renal [74] or hepatobiliary pathways [75]. Their in vivo fate, however, is not determined by composition alone. Particle size and surface chemistry, particularly surface charge, can strongly influence protein corona formation, cellular uptake, and clearance [76]. Biomimetic nanoparticles offer an additional advantage in this respect, as their surface can reproduce selected features of natural cell membranes, helping the nanocarrier evade immune recognition and prolong its circulation [77]. These biological advantages come at the cost of greater complexity in terms of preparation, reproducibility, and standardization [78].

Inorganic and carbon-based nanoparticles, on the other hand, offer more distinctive optical, magnetic, thermal, and catalytic properties, but the persistence of poorly degradable components may raise concerns regarding tissue accumulation and long-term clearance. CDs represent an interesting exception among carbon-based nanomaterials. Their ultrasmall dimensions, typically below 10 nm, may favor renal elimination and distinguish them from conventional semiconductor QDs, whose composition may include potentially toxic heavy metals [79]. Nevertheless, the biological fate of CDs is highly dependent on their physicochemical characteristics. Surface passivation and functionalization can increase their hydrodynamic size and alter their interactions with serum proteins and phagocytic cells, shifting their biodistribution and potentially favoring hepatobiliary clearance [80].

Injectable hydrogels represent a different scenario: rather than relying on systemic circulation, their main advantage lies in local administration and prolonged retention at the tumor site, which may reduce systemic exposure while maintaining the therapeutic payload close to the lesion.

The differences among these platforms become even more apparent when their imaging capabilities are considered. For several inorganic and carbon-based nanomaterials, diagnostic properties can arise directly from the material itself. QDs and CDs, for example, can exhibit intrinsic fluorescence, while the incorporation or doping of paramagnetic elements such as Mn or Fe can introduce MRI contrast capabilities. Likewise, materials with strong optical absorption and efficient photothermal conversion can support PAI, linking their diagnostic properties to the same physicochemical features exploited for therapy [81]. In contrast, most lipid- and polymer-based nanoparticles lack intrinsic imaging properties and generally require the incorporation or conjugation of fluorescent dyes, PSs, or other contrast agents. Biomimetic platforms are similarly dependent on the imaging properties of the underlying nanocarrier or encapsulated payload. From a design perspective, this distinction is relevant because nanomaterials with intrinsic imaging properties can reduce the need to introduce additional components into an already complex multifunctional system.

A different situation emerges when these platforms are considered as drug delivery systems. Organic nanoparticles are particularly versatile in this regard, as their composition can be tailored to incorporate therapeutic molecules with different physicochemical properties while enabling controlled or stimulus-responsive release [82]. Biomimetic systems can preserve this loading versatility while adding membrane-derived biological functions that favor interactions with tumor cells and the surrounding microenvironment. Carbon-based materials and porous inorganic nanostructures can also integrate therapeutic agents through mechanisms such as surface adsorption, π–π interactions, pore loading, or chemical conjugation [83]. An important difference, however, is that these materials can simultaneously contribute to intrinsic optical, thermal, magnetic, or catalytic functions, meaning that the carrier itself may actively participate in diagnosis or therapy rather than serving exclusively as a vehicle for the therapeutic payload. Hydrogels are particularly attractive when prolonged local drug delivery is required, an approach that is especially relevant to OSCC given the anatomical accessibility of the oral cavity.

Taking together, these differences make it difficult to identify a single nanoplatform that is universally superior for OSCC. Rather, the choice of material requires a balance between biocompatibility and clearance, drug-loading requirements, and the type of diagnostic information that the system is expected to provide.

## 6. Clinical Translation and Current Challenges

Despite the promising results reported for several nanoplatforms in OSCC, their translation from preclinical research to clinical testing remains challenging. This becomes particularly evident when these emerging systems are compared with PSs that have already reached clinical use or evaluation. The efficacy of PDT demonstrated in preclinical and clinical studies has supported the development of different PSs for the management of selected early-stage, superficial, or recurrent oral and head and neck tumors [84].

One example is temoporfin (mTHPC), commercially available as Foscan^®^, which has been approved in Europe for the palliative treatment of patients with advanced HNSCC who have failed previous therapies and are unsuitable for radiotherapy, surgery, or systemic chemotherapy. Temoporfin is administered by slow intravenous infusion, followed 96 h later by local irradiation at 652 nm, using a light dose of 20 J/cm^2^ delivered over approximately 200 s. Compared with several other PSs, mTHPC can be activated at relatively low drug and light doses, although prolonged cutaneous photosensitivity remains an important drawback [85]. Another widely investigated photosensitizing approach is based on 5-ALA, a precursor of the endogenous photosensitizer protoporphyrin IX. In the United States, 5-ALA is available in formulations such as Levulan^®^ Kerastick^®^ and Ameluz^®^, which are approved for dermatological indications, particularly actinic keratosis. Its application in the oral cavity, however, remains less standardized. Clinical studies have explored different 5-ALA concentrations, incubation times, light sources, irradiation parameters, and treatment schedules, making it difficult to establish a uniform therapeutic protocol. In addition, the high hydrophilicity of 5-ALA limits its penetration and retention within oral tissues, which represents a particular concern when treating lesions extending beyond the superficial epithelial layers [86].

These limitations also illustrate some of the broader challenges associated with PDT in OSCC. Effective treatment requires sufficient PS accumulation within the tumor, together with adequate light delivery and oxygen availability. Photobleaching during irradiation may further reduce PS activity [87]. Although red light around 635 nm is commonly used for PDT because of its more favorable tissue penetration compared with shorter wavelengths, penetration remains limited, making superficial and readily accessible lesions more suitable for treatment than deeply infiltrating tumors [88]. This issue is particularly relevant in OSCC, where tumor thickness and anatomical location can result in heterogeneous light exposure. At the same time, the accessibility of the oral cavity may represent an advantage over less accessible tumor sites, as it facilitates direct irradiation of selected lesions. The effectiveness of PDT is also strongly influenced by tumor oxygenation. Like many solid tumors, OSCC frequently develops a hypoxic microenvironment, in which oxygen availability is reduced compared with normal tissues. Because molecular oxygen is required for the generation of cytotoxic ROS during conventional PDT, tumor hypoxia can substantially compromise treatment efficacy [32]. Improving PS delivery, tumor selectivity, light penetration, and oxygen availability therefore remains an important goal in the development of more effective phototherapeutic strategies for OSCC.

Alongside PSs, targeted and immune-based therapies also play an important role in the treatment of OSCC. Cetuximab, together with immune checkpoint inhibitors such as the anti-PD-1 antibody nivolumab, has expanded the therapeutic options available for patients with advanced or recurrent diseases. However, the clinical benefit of these treatments remains limited. Cetuximab provides only modest survival advantage in many patients, while the efficacy of PD-1 blockade is restricted to a subset of cases and can be accompanied by immune-related adverse events. These limitations, together with the development of treatment resistance, continue to support the research for more selective and effective therapeutic strategies [11].

While nanotheranostic platforms may help overcome some of these limitations through more selective delivery and the integration of therapeutic and imaging functions, their clinical translation introduces additional challenges. In particular, toxicity, biodistribution, biodegradation, and clearance remain important concerns, as the biological fate of nanomaterials is strongly influenced by their composition and physicochemical properties. Moreover, the increasing complexity of multifunctional systems may affect reproducibility and large-scale manufacturing, highlighting the need to balance technological sophistication with clinical feasibility.

## 7. Conclusions

The development of nanotheranostic platforms represents a promising strategy for addressing some of the major clinical limitations associated with OSCC, including insufficient tumor selectivity, systemic toxicity, therapeutic resistance, and the difficulty of accurately monitoring treatment response. By integrating diagnostic and therapeutic functions within a single platform, nanotheranostics offers the possibility of combining targeted drug delivery with imaging, stimuli-responsive behavior, and multimodal therapeutic approaches.

As highlighted throughout this review, a broad range of nanoplatforms, including lipid-based, polymeric, inorganic, carbon-based, biomimetic, and hybrid hydrogel systems, have been investigated for OSCC treatment. Despite their different compositions and mechanisms of action, these platforms share the ability to integrate complementary functionalities that can improve tumor accumulation, promote controlled therapeutic activation, and enhance antitumor efficacy. In particular, the incorporation of PTT and PDT has emerged as a valuable strategy for exploiting light-responsive mechanisms and achieving synergistic effects when combined with chemotherapy, immunotherapy, molecular targeted therapy, or catalytic approaches. Moreover, the integration of imaging modalities such as fluorescence, photoacoustic imaging, MRI, and CT provides an additional dimension, enabling the assessment of nanoplatform biodistribution and tumor accumulation and, in selected cases, the longitudinal monitoring of therapeutic response.

However, despite encouraging preclinical results, the clinical translation of these systems remains challenging. Their increasing structural and functional complexity may introduce difficulties related to large-scale manufacturing, reproducibility, long-term biocompatibility, pharmacokinetics, and regulatory approval. Furthermore, many studies still rely on simplified preclinical models that do not fully reproduce the biological heterogeneity and complexity of human OSCC. Future research should therefore focus not only on increasing nanoplatform multifunctionality, but also on developing simpler, reproducible, and clinically translatable systems whose added complexity provides a clear therapeutic or diagnostic benefit. Greater use of clinically relevant models, together with standardized evaluation of safety, biodistribution, therapeutic efficacy, and imaging performance, will be essential to bridge the gap between preclinical development and clinical application.

Overall, the convergence of nanomedicine, diagnostic imaging, and multimodal therapy provides a compelling framework for the development of more precise and personalized strategies for OSCC. Rather than simply increasing the number of functionalities incorporated into a single platform, the future of OSCC nanotheranostics will depend on achieving a rational balance between multifunctionality, therapeutic efficacy, safety, and translational feasibility. Such an approach may ultimately facilitate the transition from highly sophisticated experimental nanosystems to clinically relevant tools for image-guided precision treatment of OSCC.

## Figures and Tables

**Table 4:** Summary of hybrid hydrogels investigated for theranostic applications in OSCC.

Nanoplatform	Imaging	Therapeutic Strategy	Targeting	OSCC Model/Administration	Main Findings/Limitations
CS/HEC hydrogel loaded with benzo- thiadiazole-based photothermal nanoparticles [65]	-	PTT (808 nm, 0.5 W/cm^2^, 10 min)	Local intratumoral administration	CAL-27/SCC-25 cells; orthotopic CAL-27 tongue model; i.t.	Effective photothermal tumor suppression in an orthotopic OSCC model
Thermo sensitive PDLLA-PEG-PDLLA hydrogel loaded with GA micelles (GA-MIC-GEL) [66]	-	Chemotherapy/Immunomodulation	Local intratumoral administration (in situ gelation)	SCC-7 cells; SCC-7 xenograft; i.t.	Sustained local GA delivery induced both local and systemic antitumor immunity

**Table 5:** Summary of biomimetic nanoplatforms investigated for theranostic applications in OSCC.

Nanoplatform	Imaging	Therapeutic Strategy	Targeting	OSCC Model/Administration	Main Findings/Limitations
Cancer cell membrane-coated PLGA NPs (lip-CC@ DOXNPs) [69]	FI (ICG/Cy5.5)	Chemo-PTT (808 nm, 1.5 W/cm^2^, 5 min)	Homologous targeting mediated by OSCC cell membrane	HSC-3 cells; subcutaneous and orthotopic HSC-3 xenografts; i.v.	Enhanced tumor accumulation and chemo-PTT efficacy
Photoactive neutrophils (PANs) [70]	FI	Mitochondria-targeted PDT (660 nm, 100 mW/cm^2^, 5 min)	Neutrophil chemotaxis + αvβ3 integrin targeting through RGD	CAL-27 cells; 4-NQO-induced orthotopic OSCC model; i.v.	Inflammation-driven tumor targeting and near-complete tumor eradication; scalability limited by primary neutrophil engineering
OSCC-derived exosomes (ICG@ EXOs) [71]	FI	Combined PTT/PDT + molecular targeted therapy (Gefitinib, 785 nm, (0.5 W cm^2^, 5 min)	Homologous exosome-mediated targeting	SCC-7/FaDu cells; in vitro only	Synergistic therapy suppressed primary tumors and lymph node metastasis

**Table 6:** Cross-platform comparison of the main nanoplatforms investigated for OSCC according to their biocompatibility, biodegradability and clearance, intrinsic imaging capability, and drug-loading versatility. Ratings provide a qualitative assessment of the general properties of each material class: +++, high; ++, moderate; +, low; −, absent or negligible.

Nanoplatform	Biocompatibility	Biodegradability/Clearance	Intrinsic Imaging Capabilities	Drug-Loading Versatility
Organic nanoparticles	+++	+++	+	+++
Inorganic nanoparticles	++	+	+++	++
Carbon-based nanomaterials	++	+/++	+++	++
Biomimetic nanoparticles	+++	++/+++	+	+++
Injectable hydrogels	+++	+++	−/+	+++

## Data Availability

No new data were created or analyzed in this study. Data sharing is not applicable to this article.
